# Targeted metagenomic sequencing data of human gut microbiota associated with *Blastocystis* colonization

**DOI:** 10.1038/sdata.2017.81

**Published:** 2017-06-27

**Authors:** Léa Siegwald, Christophe Audebert, Gaël Even, Eric Viscogliosi, Ségolène Caboche, Magali Chabé

**Affiliations:** 1 GENES DIFFUSION, Douai 59501, France; 2 PEGASE-Biosciences, Institut Pasteur de Lille, Lille 59019, France; 3 CRIStAL (UMR CNRS 9189 Université de Lille, Centre de Recherche en Informatique, Signal et Automatique de Lille) & Inria, Villeneuve d'Ascq 59655, France; 4 Univ. Lille, CNRS, Inserm, CHU de Lille, Institut Pasteur de Lille, U1019—UMR 8204—CIIL—Centre d’Infection et d’Immunité de Lille, Lille 59019, France

**Keywords:** Metagenomics, Parasitology, DNA sequencing, Gastrointestinal diseases

## Abstract

In the past decade, metagenomics studies have become widespread due to the arrival of second-generation sequencing platforms characterized by low costs, high throughput and short read lengths. Today, although benchtop sequencers are considered to be accurate platforms to deliver data for targeted metagenomics studies, the limiting factor has become the analysis of these data. In a previous paper, we performed an Ion Torrent PGM 16S rDNA gene sequencing of faecal DNAs from 48 *Blastocystis*-colonized patients and 48 *Blastocystis*-negative subjects, in order to decipher the impact of this widespread protist on gut microbiota composition and diversity. We report here on the Ion Torrent targeted metagenomic sequencing and analysis of these 96 human faecal samples, and the complete datasets from raw to analysed data. We also provide the key steps of the bioinformatic analyses, from library preparation to data filtering and OTUs tables generation. This data represents a valuable resource for the scientific community, enabling re-processing of these targeted metagenomic datasets through various pipelines and a comparative evaluation of microbiota analysis methods.

## Background & Summary

In recent years, the advent of high-throughput sequencing (HTS)-based metagenomics and the development of related bioinformatics approaches have greatly facilitated the study of microbial communities in humans. For example, it has allowed deeper insight into the critical role played by the community of microorganisms inhabiting our gastrointestinal tract in determining human health and disease. From a technological viewpoint, shotgun metagenomics still remains very expensive, and data analysis remains a challenging issue, due to both the size and complex structure of the data^1^. Thanks to the recent benchtop sequencer releases (Ion Torrent and MiSeq technologies), targeted metagenomics that focuses only on an informative genomic marker like 16S rDNA, has become widespread in microbiology studies, and is commonly used today to assess the gut microbiota.

Besides bacteria, which are the most numerous organisms, diverse eukaryotes, such as protists, also inhabit the human gut. Among all the human intestinal protists, some are difficult to define unequivocally as pathogens or commensals^2^. Indeed, pathogenicity is undoubtedly the rule for *Cryptosporidium* spp. and *Entamoeba histolytica*, but the clinical significance of other intestinal protists like *Blastocystis* spp. remains undetermined^2,3^.

Since their discovery, *Blastocystis* spp., reported to be the most common unicellular eukaryotes detected in human faecal samples, have generally been investigated under conditions in which they were assumed to cause disease. However, they are also detected with high prevalence in healthy people, and it has been shown that they can colonize the healthy human gut for long periods of time without resulting in symptomatic carrier status^4^. In order to address the issue of the clinical significance of these intestinal protists, studying the impact of their colonization on gut microbiota began to attract interest from the scientific community^3, 5,6,7^.

In a previous paper, we investigated the impact of *Blastocystis* colonization on the composition and diversity of the human gut microbiota by comparing the microbiota of *Blastocystis*-colonized patients and *Blastocystis*-negative individuals^3^. This study suggested that colonization by this protist is usually associated with a healthy gut microbiota, rather than with the gut dysbiosis generally observed in metabolic or infectious inflammatory diseases of the lower gastrointestinal tract^3^. This result was obtained from a cross-sectional study including two groups of 48 subjects, colonized or not by *Blastocystis*, and comparable in terms of clinical and environmental variables. Ion Torrent 16S rDNA gene sequencing of faecal DNAs was performed, followed by bioinformatics analyses to profile and compare the gut bacterial communities of these two groups.

**Figure 1:**
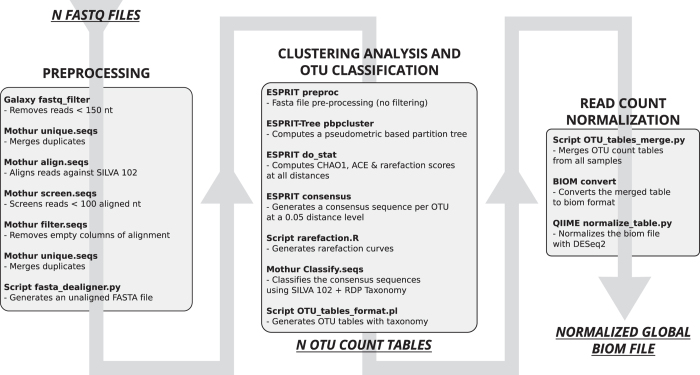
Schematic representation of the home-made bioinformatics pipeline for the analysis of targeted metagenomic Ion Torrent sequencing data, that included several publicly available tools (e.g. Mothur^10^, EspritTree^12^, QIIME^17^ or DESeq2^16^), databases (the Silva small subunit RNA database^11^ and Ribosomal Database Project (RDP)^13^) and home-made Perl/Python scripts.

We here provide detailed descriptions of DNA sample processing, including DNA extraction, quantification and monitoring, sequencing library creation, and sequencing procedures. Moreover, we report the Ion Torrent raw sequencing data (Data Citation 1), and provide a full description of the bioinformatics pipeline we used for the analysis (Fig. 1). The datasets provided here represent a valuable resource for the scientific community, enabling the re-processing of the data and a comparative evaluation of other targeted metagenomic analysis pipelines. They include: (i) the raw sequencing data (Data Citation 1) (ii) the intermediate files from our home-made 16S targeted metagenomic analysis pipeline (i.e., the OTU count tables (OTU_count_tables tsv files, Data Citation 2) and the BIOM file before data normalization (raw_global BIOM file, Data Citation 2), and (iii) the final BIOM file (normalized_global BIOM file, Data Citation 2). The intermediate files allow the scientific community to monitor, evaluate and/or optimize certain analytical steps, whereas the final BIOM file (normalized_global BIOM file, Data Citation 2) allows the confirmation of our biological results, particularly the higher bacterial diversity in faecal microbiota of *Blastocystis* colonized patients^3^, as well as a comparison of the available tools for secondary analysis of targeted metagenomic data. Finally, the release of all these datasets will be useful for the scientific community to compare microbial patterns for a variety of case-control studies.

## Methods

The patient sampling, *Blastocystis* diagnosis by real-time PCR and additional methods presented here are expanded from the descriptions in our previous paper^3^.

Briefly, in order to avoid selection bias in the choice of samples, statistical analyses were performed on patients’ clinical and environmental variables to obtain a ranked *Blastocystis* colonization risk score for each patient. The risk scores were then used to select the 24 highest score values and the 24 lowest scores in each population of *Blastocystis*-positive and *Blastocystis*-negative patients, thus determining 4 groups of 24 patients. Groups 1 and 2 were composed of *Blastocystis*-colonized patients and groups 3 and 4 included *Blastocystis*-negative patients. Groups 1 and 3 were linked in terms of low environmental and clinical risk factors, and groups 2 and 4 shared high risk factors (see Supplementary File 1).

### DNA extraction

For each subject, approx. 100 mg of fresh stools was collected and homogenized by shaking in 1.5 ml of Stool Transport and Recovery (S.T.A.R.) buffer (Roche Diagnostics, Indianapolis, IN) (ratio 1:3 according to the manufacturer’s recommendations). The stool samples stored in the S.T.A.R. buffer were centrifuged for 1 min at 1,000×g. Total genomic DNA was extracted from 200 μl of the supernatant using the QIAamp DNA Stool Mini Kit (Qiagen, Hilden, Germany) according to the manufacturer’s recommended procedure. The DNA was eluted in 100 μl of AE elution buffer (Qiagen) and stored at −20 °C until use.

### DNA extraction monitoring

Faecal DNA samples corresponding to 48 *Blastocystis*-positive and 48 *Blastocystis*-negative subjects were randomly distributed in a 96-well microplate. The total DNA concentration of each DNA extract was monitored using the Quant-iT PicoGreen dsDNA assay (Invitrogen). The 16S gene DNA copy number was assessed by a SybrGreen quantitative PCR method adapted from Maeda *et al.*,^8^ allowing both inhibition effect estimation and DNA concentration adjustment. The reaction mixture (15 μl) for the SybrGreen assay performed in RotorGene (Corbett Life Science) contained 2X Brilliant III SybrGreen qPCR Mastermix (Stratagene), primers ( GTGSTGCAYGGYTGTCGTCA, Univ16S_1048-1067 as the forward primer and ACGTCRTCCMCACCTTCCTC, Univ16S_1175_1194 as the reverse primer) with a final concentration of 560 nM and 2 μl of DNA extract as the template. The amplification conditions were 3 min at 94 °C, 45 cycles of 15 s at 94 °C for denaturation, 22 s at 60 °C for annealing and extension, followed by a melting curve from 54 °C to 95 °C with 0.5 °C increments (10 s).

### HTS library preparation

The sequence regions of the 16S rRNA gene-spanning variable regions V3-V5 were then amplified with a fusion PCR using the broad-range forward primer For16S_519, CAGCMGCCGCGGTAATAC and the reverse primer Rev16S_926, CCGTCAATTCMTTTGAGTTT (see Supplementary File 2).

Library preparations for amplicon sequencing were performed in a final volume of 100 μl containing 1X PCR buffer, 2 mM MgSO4, 1 U of DNA High-Fidelity Taq Polymerase (Invitrogen), 625 nM of each barcoded primer (IDT), 250 μM of each dNTP (Invitrogen), and a concentration-adjusted DNA sample. Each sample was taken following two PCRs, one with the sequencing adapter linked to the forward primer, and the other with the sequencing adapter linked to the reverse primer. The two resulting PCR products were equimolarly pooled after DNA purification with NucleoFast 96 PCR (Macherey Nagel), followed by a Quant-iT PicoGreen ds DNA quantification (Invitrogen).

### HTS template preparation and sequencing

Before emulsion PCR (*em*PCR), the library concentration was adjusted to 26 pM. Template preparation resulted from an emPCR in Ion 400 Template OT2 following a purification with Ion Torrent OneTouch ES according to the manufacturer's protocol.

The 96-barcoded bidirectional library was sequenced through PGM, Ion Torrent (Life Technologies) with the Ion 318 Chip and Ion PGM 400 Sequencing Kit (Life Technologies), according to the recommended protocol.

### Sequence-based microbiota analysis

The analysis of all samples was performed using a home-made Galaxy (v1.0.0)^9^ pipeline (http://www.pegase-biosciences.com/pub_2014/#ECCB), as shown in Fig. 1, linking three main analytical steps: raw data preprocessing, clustering analysis and OTU classification, and read count normalization. Unless otherwise mentioned in Fig. 1, default parameters were used for all software programs. The home-made scripts are available in Figshare (scripts.zip file, Data Citation 2).

The preprocessing step (Fig. 1), using Mothur (v1.27.0)^10^ and home-made scripts (scripts.zip file, Data Citation 2), filtered the raw data (Data Citation 1) to minimize erroneous reads generated by the Ion Torrent PGM sequencer. Reads shorter than 150 bases and/or containing large homopolymers were removed. The reads were then aligned against the SILVA 102 bacterial database^11^, and those reads with alignments of fewer than 100 bases were filtered out. Finally, the filtered reads were deduplicated to reduce the datasets. Three of the 96 samples, considered to be outliers (see the Technical validation section) were discarded in this step, before proceeding with the subsequent analyses. The number of reads remaining after this preprocessing step was 2,742,108.

In the second analytical step, OTU clustering was performed using ESPRIT-Tree^12^ version 11152011, which allows the same OTU definition precision as standard hierarchical clustering procedures, but requires less execution time. The OTUs were classified using classify.seqs in Mothur (v1.27.0)^10^ with the SILVA 102 database^11^ and the RDP taxonomy^13^ (Fig. 1).

Intra-sample rarefaction curves were generated using the home-made rarefaction curve plotting tool (rarefaction.R in scripts.zip file, Data Citation 2). It provides a way of comparing the richness observed in the samples. Graphically it presents the number of OTUs theoretically observed for a range number of sequences into the sample at variable distances (91%, 93%, 95%, 97%).

For each sample, the output of this second analytical step is an OTU table file (OTU_count_tables tsv file, Data Citation 2) containing four columns: the first column is the consensus read name associated to the OTU, the second column is the OTU raw counts, the third column is the consensus read name (same as the first column) and the fourth column is the associated taxon. The consensus sequence for a given OTU is the most abundant sequence in this OTU. The characteristics of these OTU_count_tables files are summarized in Table 1.

In the third analytical step of the home-made pipeline (Fig. 1), all the annotated OTU tables were merged into a global OTU table using a home-made python script (v2.7.3) (OTU_tables_merge.py script from scripts.zip file, Data Citation 2), based on each OTU's taxonomic annotation.

The Global OTU table is a tabulation-formatted file (TSV) in which each column of this table represents one sample, and each line represents one taxon (identified by its OTU identifier in the first column and by the taxonomic annotation in the last column). This merged OTU table describes 474 OTUs, their annotation, and the number of reads belonging to each OTU per sample. Finally, this annotated OTU table was converted into a global BIOM file^14^ by the biom (v2.1.4) convert command. HDF5 format was chosen to optimize the storage. The characteristics of the BIOM file (Data Citation 2) are summarized in Table 2.

As advised by previous recommendations^15^, the DESeq2 package^16^ integrated into QIIME (v1.9.0)^17^, was used to normalize the total read counts and avoid rarefaction of the read count data. The normalization_table.py python script from QIIME (v1.9.0) was configured with the following options: the algorithm chosen was DESeq2, replacing negative numbers produced by the DESeq normalization technique with zeros. A normalized Global BIOM file was then produced (Table 3). Note that the taxonomic information disappears after this normalization step. The biom (v2.1.4) 'add-metadata' option was used to add the taxonomic information back and obtain a fully annotated and normalized BIOM file (Data Citation 2).

### Ethical approval

Ethical approval surrounding the procurement of samples used in this study was obtained from the Research Ethics Committee ‘Comité de Protection des Personnes Sud-Est 6, France’ (reference number 2015/CE82), which waived the requirement for informed consent because the experiments did not result in additional constraints for the patients. All the methods were carried out in accordance with the approved guidelines (World Medical Association’s (WMA) Declaration of Helsinki-Ethical Principles for Medical Research Involving Human Subjects).

## Data Records

The raw data from each of the 96 sequenced samples were submitted to the Sequence Read Archive (SRA) of the NCBI, under Project ID PRJNA342805 (Data Citation 1). The raw data were deposited under each barcode index. The association between the sample name, sample ID (index), and *Blastocystis* colonization status and group is available in Supplementary File 1.

The data analyzed using the home-made metagenomics pipeline (Fig. 1) are available on the PEGASE-biosciences website: http://www.pegase-biosciences.com/collaborations/blastocystis-gut-microbiota/.

The taxonomic assignment before data normalization is available per sample in the OTU_count_tables tsv files (Data Citation 2) and the raw_global.biom file (Data Citation 2); and after data normalization (process described in the Methods section) in the normalized_global.biom file (Data Citation 2).

Finally, the four home-made scripts used in the bioinformatics pipeline (Fig. 1) are available in the scripts.zip file (Data Citation 2).

## Technical Validation

### Data quality check

**Figure 2:**
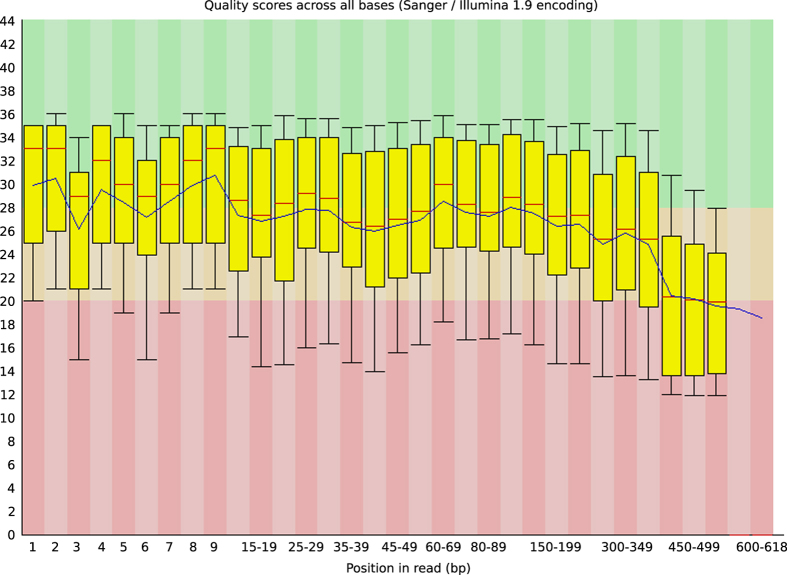
Quality scores across all bases box-and-whiskers plot (FastQC Read Quality reports (Galaxy Version 0.67)). Red line=median value, blue line=mean value, yellow box=inter-quartile range, upper and lower whiskers = 10% and 90% points respectively.

As shown in Fig. 2, the quality scores of the raw Ion Torrent reads (average Q27) are quite stable across the reads, above the Q20 reported accuracy for Ion Torrent PGM sequencing. Even the ends of the reads, which are known to be of lesser quality, are still within the Q20 range. This allowed the use of the complete reads for downstream analyses, without requiring a quality read trimming step.

### Raw data output

A total of 3,962,103 reads were obtained from the Ion 318 Chip sequencing run of the 96 indexed samples. As expected, a length mode of approximately 400 bases (403 bases) was observed, with an average read number per index of 41,562 and an average read length of 272 bases. These metrics assessed the good quality/quantity of the generated raw data (Table 4).

Three of the 96 samples were discarded after sequencing (see Supplementary File 1). Indexes 50 and 63 were considered to be outliers due to the low number of reads (<10,000) for these samples. A third outlier (index 18) was due to an accidental handling error and was also discarded from the data set.

ANOVA tests were conducted to compare the read number per index and the read lengths from the output sequence data in the four groups of patients. There were no significant differences between the four groups for any variable, indicating that there was no technical bias in sequencing for the four groups of patients (mean read length per group (*P*-value=0.47) and mean read number per group (*P*-value=0.78)) (Table 5).

## Additional Information

**How to cite this article:** Siegwald, L. *et al.* Targeted metagenomic sequencing data of human gut microbiota associated with *Blastocystis* colonization. *Sci. Data* 4:170081 doi: 10.1038/sdata.2017.81 (2017).

**Publisher’s note:** Springer Nature remains neutral with regard to jurisdictional claims in published maps and institutional affiliations.

## Supplementary Material



Supplementary File 1

Supplementary File 2

## Figures and Tables

**Table 1:** OTU_count_tables files (Data Citation 2) summary.

**Origin**	**Type**	**Number of files**	**Number of samples per file**	**Mean read number per raw OTU and s.e.**	**Mean number of unique taxa**	**Mean ratio of unique taxa to total number of taxa**
Home-made Galaxy pipeline	TSV	93	1	25 (s.d.: 258.6)	1,184 (s.d.: 846)	0.77

**Table 2:** Raw Global BIOM file (Data Citation 2) summary.

**Origin**	**Type**	**Number of samples**	**Number of observations (OTU)**	**Total read count**	**Table density (fraction of non-zero values)**	**Mean read count per sample**	**Median read count per sample**	**Additional column: observation metadata categories**
Global OTU table	Biom	93	474	2,742,108	0.187	29,485 (s.d.: 5,739)	29,419	Taxonomy

**Table 3:** Normalized Global BIOM file (Data Citation 2) summary.

**Origin**	**Type**	**Number of samples**	**Number of observations (OTU)**	**Total count**	**Table density (fraction of non-zero values)**	**Mean counts per sample**	**Median counts per sample**	**Additional column: observation metadata categories**
Global OTU table—biom format	Biom	93	405	33,624	0.217	361 (s.d.:138)	353	Taxonomy

**Table 4:** Read metrics from output PGM quality-approved, trimmed and filtered sequence data.

	**Mean read length (bases)**	**Median read length (bases)**	**Mean read number per index**	**Median read number per index**	**Min. read number per index**
Raw data*	272.67	273	42,603	41,956	14,617

*Three outlier samples (indexes 18, 63 and 50 belonging to groups 1, 2 and 3 respectively) were discarded before the analyses.

**Table 5:** Mean read length and read number per index from output PGM quality-approved, trimmed and filtered sequence data in the four groups of patients.

	***Blastocystis*** **sp.-positive**		***Blastocystis*** **sp.-negative**	
	**Group 1**	**Group 2**	**Group 3**	**Group 4**
Mean read number per index*	43,058	44,150.04	41,374.65	41,862.54
Mean read length (bases)*	270	273.96	275.13	271.62

*Three outlier samples (indexes 18, 63 and 50 belonging to groups 1, 2 and 3 respectively), were discarded before the analyses.
